# Assaying RNA structure with LASER-Seq

**DOI:** 10.1093/nar/gky1172

**Published:** 2018-11-22

**Authors:** Boris Zinshteyn, Dalen Chan, Whitney England, Chao Feng, Rachel Green, Robert C Spitale

**Affiliations:** 1 Department of Molecular Biology and Genetics, Johns Hopkins University. Baltimore, MD 21205, USA; 2 Department of Pharmaceutical Sciences, University of California, Irvine, Irvine, CA 92697, USA; 3 Howard Hughes Medical Institute, Johns Hopkins University School of Medicine, Baltimore, MD 21205, USA; 4 Department of Chemistry, University of California, Irvine, Irvine, CA 92697, USA

## Abstract

Chemical probing methods are crucial to our understanding of the structure and function of RNA molecules. The majority of chemical methods used to probe RNA structure report on Watson–Crick pairing, but tertiary structure parameters such as solvent accessibility can provide an additional layer of structural information, particularly in RNA-protein complexes. Herein we report the development of Light Activated Structural Examination of RNA by high-throughput sequencing, or LASER-Seq, for measuring RNA structure in cells with deep sequencing. LASER relies on a light-generated nicotinoyl nitrenium ion to form covalent adducts with the C8 position of adenosine and guanosine. Reactivity is governed by the accessibility of C8 to the light-generated probe. We compare structure probing by RT-stop and mutational profiling (MaP), demonstrating that LASER can be integrated with both platforms for RNA structure analyses. We find that LASER reactivity correlates with solvent accessibility across the entire ribosome, and that LASER can be used to rapidly survey for ligand binding sites in an unbiased fashion. LASER has a particular advantage in this last application, as it readily modifies paired nucleotides, enabling the identification of binding sites and conformational changes in highly structured RNA.

## INTRODUCTION

RNA molecules play essential roles in nearly every step of gene regulation, from chromatin modification and transcription to translation regulation. RNA molecules fold into complex three-dimensional structures that can impart unique functionalities, from phosphodiester bond cleavage to protein binding (1,2). Several existing chemical methods directly measure RNA structure, both inside and outside of living cells. Conventional chemical probes such as dimethyl sulfate (DMS, which methylates the Watson–Crick face of single-stranded adenosine and cytosine residues, as well as the 7 position of guanosine) and SHAPE (selective 2′-hydroxyl acylation analyzed by primer extension, which modifies any nucleotide by 2′-hydroxyl acylation at flexible sites) report primarily on the Watson–Crick pairing status of individual nucleotides (3–5). A critical component of the RNA structure toolbox is the ability to interrogate the surface opposite the Watson–Crick face to obtain a more general map of nucleobase solvent accessibility. Hydroxyl radical footprinting (HRF) has been used for decades to assay solvent accessibility by cleaving the sugar-phosphate backbone of the RNA at accessible nucleotides (6). While HRF is easily implemented *in vitro, in vivo* probing requires a synchrotron X-ray source (7).

**Figure 1.**
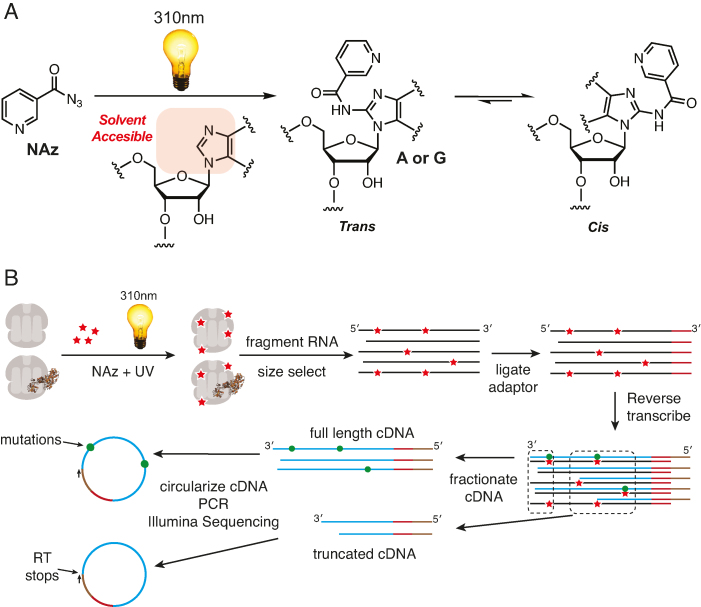
Chemical probing by LASER-seq and LASER-MaP. (**A**) Nicotinoyl azide (NAz) is activated by long-wavelength UV light to form C8 adducts on A and G residues. Adduct formation is thought to result in trans-to-cis isomerization of the nucleobase. Such isomerization provides a molecular explanation for the production of RT-stops, as observed previously with denaturing gel electrophoresis, and for nucleotide misincorporations. (**B**) LASER-Seq and LASER-MaP methods. Ribosome complexes or intact cells were treated with NAz and UV light, followed by RNA extraction, fragmentation, and size selection. After adaptor ligation and reverse transcription, cDNAs were size selected and separated into full-length and truncated products, which were separately circularized and subjected to high-throughput sequencing.

We recently reported the development of Light Activated Structural Examination of RNA, or LASER (8). LASER takes advantage of light-activated aroyl azides such as nicotinoyl azide (NAz), which can form aroyl nitrenium ions in solution. Nitrenium ion electrophiles can react with electron-rich purine residues in RNA, through an electrophilic aromatic substitution reaction, to form C8 amide products with adenosine and guanosine (Figure 1A). These C8 adducts can induce a reverse transcription stop, likely due to isomerization (*trans*-to-*cis*) along the C1′-N9 bond of adenosine and guanosine, and these RT-stops can be used to map solvent accessibility onto the primary structure of an RNA. Because the aroyl azides are readily taken up by cells, LASER can be utilized to footprint unique structural states and protein-RNA interactions within living cells.

The merging of chemical methods to measure RNA structure with deep sequencing has opened the door to large-scale analyses of RNA structure. DMS, *N*-cyclohexyl-*N*’-(2-morpholinoethyl)carbodiimide metho-*p*-toluenesulfonate (CMCT), and SHAPE have now been utilized by many labs to probe pools of RNAs or entire transcriptomes (9–19). HRF was also recently adapted for use with high-throughput sequencing (20), and we have recently modified this high-throughput technique to identify *in vitro* protein binding sites on the ribosome by localized generation of hydroxyl radicals *in situ* (21). Since hydroxyl radicals cause cleavage of the RNA backbone, they can only be identified by RT stop approaches, without the benefits of recent mutational profiling (MaP) technologies (22). MaP relies on the propensity of some covalent nucleotide modifications to cause mutations in addition to RT stops during reverse transcription, which can be quantified by high-throughput sequencing. MaP approaches have been used to identify sites of modification by dimethyl sulfate (DMS) (23,24), SHAPE reagents (22,24), and other probes that covalently modify RNA (19,25). Despite its utility in DMS and SHAPE structure probing, expansion of MaP to the many other chemical probes has yet to be realized. To expand LASER to studies of large structured RNAs, we developed LASER-Seq and LASER-Mutational Profiling (LASER-MaP). LASER reactivity should report on the solvent accessibility of the C8 position, providing an additional layer of information and allowing identification of binding sites or conformational changes in base-paired regions. Here we use the ribosome, a large ribonucleoprotein of complex but well-defined structure, as a test case for LASER-Seq and LASER-MaP. We find that LASER reactivity generally agrees with computed solvent accessibility across the ribosome, and that LASER can be used to rapidly survey the ribosome for ligand binding sites in an unbiased fashion. LASER has a particular advantage in this last application, as it readily reacts with paired nucleotides, enabling the identification of binding sites and conformational changes in highly structured RNA.

## MATERIALS AND METHODS

### Synthesis and storage of chemical reagents

NAz was synthesized as in (8), and 1M7 was synthesized as in (26). NAz was stored in powder form, wrapped in aluminum foil, at −20°C. NAz was dissolved in anhydrous DMSO at 3 M and used within a few days. 1M7 was dissolved in anhydrous DMSO immediately before use. Onc112 peptide (VDKPPYLPRPRPPR{d-ARG}IYN{d-ARG}) was synthesized by GenScript (Piscataway, NJ, USA), resuspended in water to 20 mM, and stored at −20°C.

### Ribosome and EF-G purification

Crude *Escherichia coli* 70S ribosomes (27) were isolated from strain MRE600 (ATCC29417), grown to OD600 of 0.6 in 6 l of LB with no antibiotics. Cultures were chilled on ice for 30 min, harvested by centrifugation, resuspended in buffer A (20 mM K-HEPES pH 7.5, 100 mM KCl, 10 mM MgCl_2_, 0.5 mM EDTA, 6 mM βME), and lysed in a French press. Lysates were clarified twice by centrifugation at 8°C at 22 000 RPM for 15 min in an SS-34 rotor. Ribosomes were pelleted through a 35 ml sucrose cushion (1.1 M sucrose, 20 mM K-HEPES pH 7.5, 500 mM KCl, 10 mM MgCl_2_, 0.5 mM EDTA) in Ti-45 tubes for 16 h at 17 000 rpm. After removing the cushion, ribosomal pellets were gently washed with 1 ml buffer B (20 mM K-HEPES pH 7.5, 500 mM KCl, 10 mM MgCl_2_, 0.5 mM EDTA), and gently resuspended in buffer A. His-tagged EF-G was purified as in (28). *Saccharomyces cerevisiae* strain YAS2488 (MATa leu2–3 112 his4–539 trp1 ura3–52 cup1::LEU2/PGK1pG/MFA2pG) was grown with shaking in YPD (1% yeast extract, 2% peptone, 2% glucose) at 30°C to an OD600 of 1.0 and 40S and 60S ribosomal subunits were purified exactly as in (29).

### *In vitro* NAz treatment of *E. coli* ribosomes

*In vitro* LASER reactions were performed in 25 μl volumes in 1.5 ml microcentrifuge tubes (Axygen). 25 μl reactions contained 1× HEPES modification buffer (30 mM K-HEPES pH 7.5, 7 mM Magnesium Acetate, 100 mM KCl), 1–2 μM crude *E. coli* 70S ribosomes, and where indicated 4 μM EF-G, 0.5 mM GDPNP, or onc122. For the first batch of experiments we performed 1 reaction each of 70S alone, 70S+EF-G, 70S+EF-G+GDPNP. For the second batch, we performed two reactions with 70S alone with and without NAz, and one reaction at each onc112 concentration. Reactions were brought to the final volume of 25 μl by adding 2 μl of 300 mM NAz in DMSO or DMSO alone. Reducing agents (besides those present in ribosome and protein stocks) were omitted to prevent potential reactivity with NAz. Reactions were incubated for 5 min at 37°C, arranged uniformly around a UV lamp (20 watt Zilla Desert 50 UVB Fluorescent Coil Bulb) with the tube bottoms pointed towards the bulb, and exposed to UV light for 3 min. Reactions were brought to 300 μl with 0.3 M sodium acetate pH 5.5, isopropanol precipitated, extracted twice (or until the protein interface was gone) with phenol/chloroform/isoamyl alcohol, and once with chloroform, ethanol precipitated, and resuspended in 40μl water. Precipitations (30) were performed in 1.5 ml microcentrifuge tubes by bringing solutions to 0.3 M sodium acetate, adding 5–10 μg of glycogen (if RNA is being quantified) or glycoblue (Invitrogen), and an equal volume of isopropanol or 2.5 volumes of ethanol, chilling for 15 min on dry ice, centrifuging at 20 000g for 30 min, and removing the supernatant. Pellets were washed by adding 400 μl 70% (for intact RNA) or 80% (for fragmented RNA) ice-cold ethanol, centrifuging again for 5 min, and removing supernatant.

### *In vivo* NAz treatment of K562 cells

K562 cells were grown in advanced RPMI 1640 media (Gibco) supplemented with 10% FBS (Gibco) and 2 mM l-Glutamine (Gibco). 500 000 cells were pelleted in a 1.5 ml microcentrifuge tube (Axygen), washed with 500 μl 37°C PBS, pelleted again, and resuspended in 100 μl PBS + 10% DMSO, or PBS + 300 mM NAz. One biological replicate each were exposed to UV light for 1 or 3 min as described for *in vitro* reactions. 300 μl of Trizol (Invitrogen) was added to each reaction and RNA was purified with the Zymo Direct-Zol RNA miniprep kit, following the manufacturer's directions for total RNA extraction, omitting the DNase treatment.

### *In vitro* NAz, 1M7 and BzCN treatment of *S. cerevisiae* ribosomes

**Figure 2.**
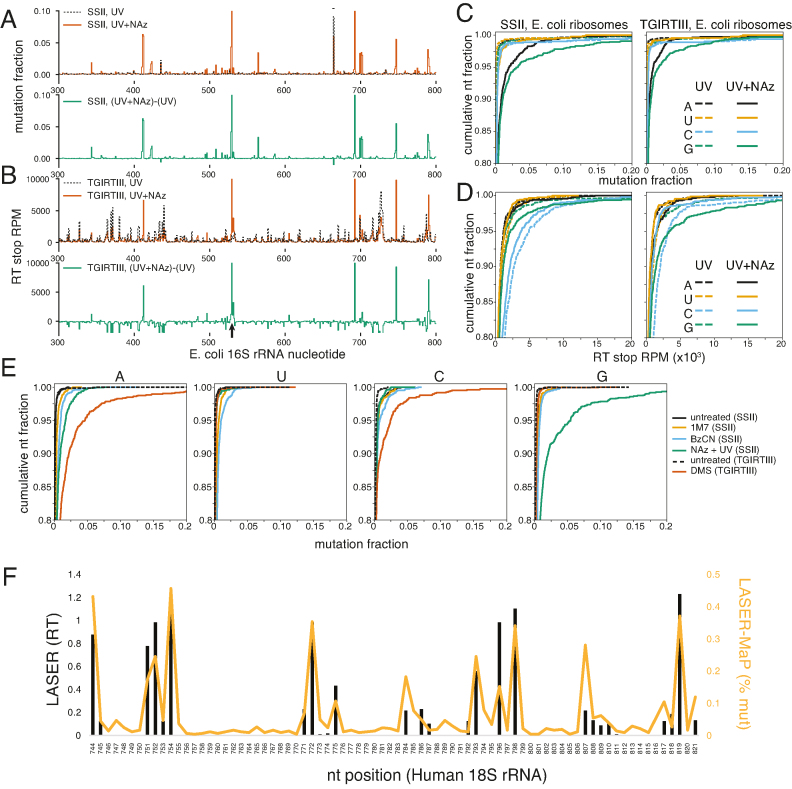
LASER-Seq detects RT stops and LASER-MaP detects mutations from NAz modification. (**A**) LASER-MaP Mutation fraction (for SSII) or (**B**) LASER-Seq RT stop RPMs (for TGIRTIII) for a section of the *E. coli* 16S rRNA from libraries prepared with or without NAz. The background-subtracted difference plots for these same traces are presented below each plot in green. A single nucleotide indel between genomic rRNA copies at position 671 is indicated by -. Nucleotide G530 is indicated with an arrow. (**C**) Cumulative distributions of mutation rates, separated by nucleotide identity, for LASER-MaP libraries made with or without NAz. The y axis indicates the fraction of nucleotides with a mutation rate not exceeding the given threshold (x axis). Note that axes are truncated to only show the upper-left corner of the distribution, and the full range of both axes is 0–1. (**D**) Cumulative distributions of RT stop RPMs for LASER-Seq libraries made with or without NAz. (**E**) Cumulative distributions of MaP mutation rates for *S. cerevisiae* libraries probed with various reagents and reverse transcribed with SSII (1M7, BzCN, NAz) or TGIRTIII (DMS). DMS data are from (44). (**F**) Comparison between LASER-MaP mutation frequency and band intensities of manual RT-stops from LASER (8), both performed in cultured human cells.

25μl reactions were assembled containing 1× HEPES modification buffer (30 mM K-HEPES pH 7.5, 3mM Magnesium Acetate, 100 mM KCl, 2 mM DTT) and 0.5 μM each of *S. cerevisiae* 40S and 60S ribosomal subunits. After incubating at 25°C for 5 min, reactions were brought to the final volume of 25 μl by adding 2.5 μl of 100 mM 1M7, 100 mM Benzoyl Cyanide (BzCN, Sigma Aldrich 115959) or 3M NAz in anhydrous DMSO, or DMSO alone, and incubated at 25°C for 6 min (1M7), 30 s (BzCN), or exposed to UV light for 3 min (NAz). Reactions were brought to 500 μl with 0.3 M sodium acetate pH 5.5, isopropanol precipitated, resuspended in 200 μl 0.3 M sodium acetate pH 5.5, extracted twice (or until protein interface was gone) with phenol/chloroform/isoamyl alcohol, and once with chloroform, ethanol precipitated and resuspended in 40 μl water. The datasets presented in Figure 2E and Supplementary Figure S4 are each from a single reaction.

### Sequencing library preparation

Methods for library preparation have been adapted from (22,23,31). 1–4 μg of total RNA was fragmented by incubation at 95°C for 5 min with 10 mM ZnCl_2_. Fragmentation was stopped by placing tubes on ice and immediately adding EDTA to 20 mM. RNA was ethanol precipitated, resuspended in 10 mM tris pH 7.0 + 10 μl 2× RNA gel loading dye (95% formamide, 20 mM Tris, 2 mM EDTA, xylene cyanol, bromophenol blue), incubated for 3 min at 95°C to denature RNA, and run on a 10% PAGE TBE-urea gel for size selection. A section corresponding to 100–120 bp on a (non-denatured) DNA ladder was cut from the gel. The gel was shredded by forcing the gel through a needle hole in a 0.5 ml tube by centrifugation, and RNA was eluted into 400 μl water by heating at 70°C with rapid shaking for 15 min. Gel pieces were filtered out with a Spin-X column (corning) and RNA was isopropanol precipitated, resuspended in PNK mix (3.5 μl 10 mM Tris pH 8.0, 0.5 μl SUPERaseIn RNase inhibitor (Invitrogen), 0.5 μl 10× PNK buffer (NEB), 0.5 μl T4 PNK), and incubated at 37°C for 1 h. Reactions were supplemented with 5 μl ligation mix (3.8 μl 50% PEG 8000, 0.2 μl 50 μM pre-adenylated linker 2 (oBZ191) (for oligos, see Supplementary Table S1), 0.5 μl 10× T4 RNA ligase buffer (NEB), 0.5 μl T4 RNA ligase 2 truncated (NEB)) and incubated at 37°C for 3 h, then isopropanol precipitated. For *E. coli* experiments, pellets were resuspended in water and split for reverse transcription with either TGIRTIII (Ingex) or SSII (Invitrogen). For all other experiments, only SSII was used, and pellets were directly resuspended in annealing mixture. For TGIRTIII, 10 μl reactions were prepared in PCR strip tubes with RNA, 2 μl Invitrogen 5× FS buffer, 0.5 μl 10 mM dNTPs, 0.5 μl SUPERaseIn, 0.5 μl 100mM DTT, 0.5 μl 25μM RT primer oBZ192, 0.5 μl 10 μM TGIRTIII, and incubated at 60°C for 1 h. For SSII, 6 μl annealing reactions were prepared containing RNA, 0.6 μl 10× MaPBasic Buffer (500 mM Tris pH 8.0, 750 mM KCl, 100 mM DTT), 0.5 μl 25 μM RT primer oBZ192. RNA was annealed to primer by incubating in a thermal cycler: 4 min at 65°C, 2 min at 55°C, 2 min at 45°C, 2 min at 42°C, hold on ice. 6 μl of extension mix (2.2 μl water, 0.6 μl MaPBasic buffer, 0.6 μl 10 mM dNTPs, 0.5 μl SUPERaseIn, 1.44 μl 50 mM MnCl_2_, 0.6 μl SSII) was added, mixed, and incubated at 42°C for 1 h. After all RT reactions, RNA was degraded by addition of 1 μl 5 M NaOH, and incubation for 3 min at 95°C. cDNA was precipitated, resuspended in 10 mM Tris pH 7, incubated for 3 min at 95°C, and run on a 10% TBE–urea gel. For mutation datasets, full length RT products were isolated. For all RT stop datasets, including the untreated controls, cDNA was isolated from ∼20nt above the RT primer to ∼20nt below the full-length product. Gel extraction was performed as above, pellets were resuspended in Circ ligase mix (15.5 μl 10 mM Tris pH 8.0, 2 μl 10× Circ ligase buffer, 1 μl 1mM ATP, 1 μl 50 mM MnCl_2_, 0.5 μl Circ Ligase I (Epicentre)), and incubated at 60°C for 2 h, then 80°C for 20 min. Circularized cDNA was amplified with 8–12 cycles of PCR, gel purified, and subjected to Illumina sequencing on a HiSeq 2500 with 50 bp single-end reads.

### Processing of sequencing data

Raw reads were trimmed of 3′ adaptor sequence (CACTCGGGCACCAAGGAC) with skewer (32). ShapeMapper 2.0 (33) was used to trim low-quality sequences, align reads to the consensus MRE600 (34) or *S. cerevisiae* (Saccharomyces Genome Database) rRNA sequence, and count mutations and read coverage at each position. Mutation fractions were defined as the number of reads not matching the reference at a given nucleotide position, divided by the total number of reads overlapping the position. A number of rRNA positions have nucleotide modifications or vary between rRNA copies within an organism, causing a high background of apparent mutations at that position. These positions were detected by manual inspection of mutation traces in a genome browser (35,36) and excluded from downstream analysis. These positions were *E. coli* 16S rRNA 1207, 1498, 1518, 1519; *E. coli* 23S rRNA 745, 1915; *S. cerevisiae* 18S rRNA 1191, 1781, 1782; *S. cerevisiae* 25S rRNA 645, 2634, 2843; *Homo sapiens* 18S rRNA 1248, 1851; *H. sapiens* 28S rRNA 60, 1322, 3041, 3506, 4530, 4805, 4906. For RT stop analysis, trimmed reads were mapped to rRNA sequences using STAR (37), and 5′ ends were counted. Soft-clipped nucleotides were ignored when determining read ends to reduce the effect of untemplated nucleotides added during RT. For background subtraction, the RT stop RPM or mutation rate for the UV-only control was subtracted from the NAz-treated control for each nucleotide. A data analysis pipeline that performs these processing steps and outputs tables of RT stops and mutations is available on GitHub (https://github.com/borisz264/LASER_seq_2018). Raw reads and processed data have been deposited in the Gene Expression Omnibus with accession GSE113529.

### Counting mutation co-occurrences in sequencing reads

In order to identify mutations in each read, we used the ‘–output-parsed-mutations’ flag in ShapeMapper 2.0 to produce a per-read list of mutations and their positions within the rRNA. We parsed this list to count the number of mutations in each read, only counting mutations as separate if their positions were separated by five or more nucleotides in the rRNA reference.

### Detection of ligand-dependent reactivity changes

For computation of mutation rate change, fold change, or significance, background subtraction was not used. To determine significantly protected or deprotected nucleotides we performed the analysis method presented in (38) with the following minor modifications. For each nucleotide, mutation rates (M) were normalized by dividing by the average mutation rate (*A*) across all A and G nucleotides in the rRNA. The normalized mutation rate N is M/A. For each nucleotide, the difference in the normalized mutation rate between bound (*N*_b_) and unbound (*N*_u_) is Δ*N* = *N*_b_ – *N*_u_. The standard error (σ) of the mutation rate for a nucleotide is the square root of the mutation rate divided by the read depth (*C*), which is further scaled by the average mutation rate: $\sigma = (\sqrt {M/C)} /A$. *Z* factors were computed as $z = 1 - (1.96*( {{\sigma _b} + {\sigma _u}} )/| {\Delta N} |$. Standard reactivity change scores were computed as $ = \frac{{\Delta N - mean( {all\ \Delta N} )}}{{st\_dev( {all\ \Delta N} )}}$. A nucleotide was considered to have a significant reactivity change if |*S*| > 0 and *z* > 0. For MA plots, fold change were computed as *M*_b_/*M*_u_ and average read counts were computed as (*M*_u_*C*_u_ + *M*_b_*C*_b_)/2. Since LASER does not modify all nucleotides, we did not average the signal over a sliding window or require multiple affected nucleotides within a range to call a nucleotide as protected or deprotected. Since this error model only accounts for the raw error inherent in read counting, and not any biological, biochemical, or experimental noise, we limited our hits to those found in all of the datasets. We used the same control dataset for all ligands within a batch for a given RT.

### Computation of solvent-accessible surface area ROC curve analysis

To determine the solvent accessible surface area of the C8 or 2′OH positions of purines, we used the ‘get_area()’ command in PyMol (Schrödinger, LLC). We used PDB ID 4ybb for *E. coli*. and 4v88 for *S. cerevisiae* ribosomes. We set the ‘solvent_radius’ parameter to either 3, 4 or 5 Å, ‘dot_solvent’ to 1, and ‘dot_density’ to 3. Solvent accessible surface area values are reported in Å^2^. Nucleotides that were excluded from MaP quantification (see ‘processing of sequencing data’ above) or unresolved in the structure were excluded from ROC analysis. A ROC curve was generated by iterating a mutation rate threshold from 0 to 1 in steps of 0.00001 and counting the number of nucleotides below this threshold with SASA ≥ 5 Å^2^ (true positives) or SASA < 5 Å^2^ (false positives).

## RESULTS

### LASER-Seq and LASER-MaP detect solvent-accessible nucleotides *in vitro* and *in vivo*

To adapt LASER to high-throughput sequencing methodology, we performed a pilot experiment with purified *E. coli* ribosomes (Figure 1B). We equilibrated ribosomes with 300 mM NAz, or an equal volume of DMSO, and exposed the mixture to ultraviolet (UV) light for 3 min. We prepared sequencing libraries using two different reverse transcriptases (RTs) (Superscript II (SSII), and TGIRTIII) and conditions which were previously optimized to detect DMS and SHAPE modifications by MaP analysis (22,23). We gel-purified full-length cDNA for MaP analysis, and truncated cDNA to enrich for RT stops. The isolated RT products were subjected to Illumina sequencing and the resulting sequences were mapped back to the consensus ribosomal RNA (rRNA) sequence to count RT stops (5′ ends of reads) and mutations. For RT stop analysis, NAz reactivity is expressed as Reads Per Million (RPM): the number of reads with 5′ ends mapping 1nt 3′ of the nucleotide divided by the number of reads mapping to the rRNA (in millions). For MaP analysis, NAz reactivity is expressed as the number of mutations at a nucleotide position divided by the number of sequencing reads overlapping that position.

In the absence of NAz, a few prominent peaks of mutations were visible across the rRNA (Figure 2A and Supplementary Figure S1). These sites include post-transcriptionally modified nucleotides and sites of heterogeneous rRNA sequence. There is a substantial background of RT stops spread across the rRNA, presumably due to structure or sequence dependent RT stops (Figure 2B and Supplementary Figure S2). This background was reduced upon NAz treatment, as sequencing space became occupied by NAz-dependent RT stops, and was further reduced by subtracting the UV control (Figure 2B and Supplementary Figure S2). UV treatment alone caused little change in RT stops or mutations, indicating that UV-induced RNA damage is not a major source of background in this assay (Supplementary Figures S1 and S2). After combined NAz and UV treatment, mutations and RT stops became evident at many other positions. For example, 16S G530, a highly-accessible nucleotide involved in recognition of codon-anticodon pairing during translation (39), displays strong NAz-dependent peaks of mutations and RT stops (Figure 2A and B), demonstrating the strong signal over background for this technique at accessible nucleotides.

In accordance with the specificity of NAz for A and G nucleotides, LASER treatment caused an increase in mutations and RT stops at A and G (Figure 2C and D). This effect was substantially better for MaP analysis compared to RT stop analysis, where background RT stops are a clear problem. Subtraction of RT-stop background leads to a substantial improvement in detection of NAz-dependent RT stops at A and G (Supplementary Figures S2 and S3B), while the MaP data were only marginally affected by background subtraction (Supplementary Figures S1A and S3A) due to the low background mutation rate of LASER-MaP (Figure 2C, Supplementary Figure S1). SSII yielded higher rates of mutations at purines than TGIRTIII (Figure 2C), while RT stops produced by TGIRTIII were substantially more enriched for purines, indicative of the higher RT stop background with SSII (Figure 2D, Supplementary Figure S3B). This is readily visible in Supplementary Figure S2, where the 16S rRNA landscape is dominated by a small number of high-intensity RT stops for TGIRTIII, consistent with the generally low accessibility of a highly structured and protein-bound RNA, while the SSII sample has a more uniform background of RT stops resembling the untreated control. This striking difference could be due to the higher temperature of reverse transcription for TGIRTIII (60°C, compared to 42°C for SSII), which would unfold RNA structure and might reduce structure-dependent RT stops in favor of NAz-dependent ones. For these reasons, we recommend the use of TGIRTIII for LASER-seq, and the use of SSII for LASER-MaP.

We found that mutations and RT stop RPMs were reproducible within a given RT (Supplementary Figure S3C and D) and between the different RTs (Supplementary Figure S3E). RT stops were poorly correlated with mutations, but the correlation increased when the background signal was subtracted (Supplementary Figure S3F). This indicates that the two assays have different sources of background noise, but some real NAz-dependent signal can still be detected at the same nucleotides by both methods, despite obvious differences.

These results show that both LASER-Seq and LASER-MaP are capable of detecting NAz-reactive nucleotides across a structure as large as the ribosomal RNA. However, our MaP datasets clearly have fewer positions with non-specific background compared to the RT stop datasets (Compare Supplementary Figures S1 and S2). It is exciting that LASER is well-suited to MaP analysis, as modification detection methods based on RT stops suffer from RT shadowing (read coverage is reduced immediately downstream of a heavily modified base) (23) and biases in library preparation due to the fact that the sequence at the RT stop determines the efficiency of capture during circularization (40–42). MaP-based methods suffer much less from these issues, making them a potentially more accurate measure of nucleotide modification. Most importantly, for MaP analysis, the mutation fraction is internally normalized by read coverage at the position being analyzed, and can be described by a rigorous error model (22–24,38). For these reasons, we focused on LASER-MaP for further experiments.

To contextualize the mutation rates seen in LASER-MaP, we compared it to other structure probing strategies that are amenable to mutational profiling. With purified *S. cerevisiae* ribosomes as our target, we performed LASER-MaP as well as SHAPE-MaP (22) using the reagents 1-methyl-nitrosatoic anhydride (1M7) and benzoyl cyanide (BzCN). SHAPE monitors internucleotide flexibility through 2′-OH acylation and previous reports have demonstrated that SHAPE reactivity is not governed by solvent accessibility (43). We also compared these datasets to our previously published DMS-MaP probing of the same ribosomes (44). NAz produced more mutations at G than all other tested reagents, and more than the SHAPE reagents, but less than DMS, at A (Figure 2E, Supplementary Figure S4).

MaP can identify correlated mutations on RNA by identifying multiple mutations in a single sequencing read, because RT does not necessarily terminate at the first modified nucleotide. This information can be used to refine RNA tertiary structures, or to deconvolute a mixed RNA population (24). As a preliminary test of the suitability of NAz for this application, we counted the number of reads with multiple mutations, as a fraction of the whole sequencing library. We found a 1.7- to 2.5-fold enrichment in reads with two mutations, and a 3- to 5-fold enrichment in reads with three mutations in NAz-treated samples, compared to the UV control (Supplementary Figure S5). This indicates that LASER-MaP is potentially suitable for correlated probing analyses such as RING-MaP (24).

NAz is cell-permeable (8), allowing for probing to be done inside living cells. We performed LASER-MaP on live human K562 cells and compared the data to our previously published LASER RT-stop data from radioactive primer extension of ribosomes in HeLa cells (8). As shown in Figure 2F, the two methods agree with each other, further demonstrating that LASER works to modify RNA inside living cells and that LASER-MaP can recapitulate the results from manual probing of RNA.

### LASER-MaP is specific for measuring solvent accessibility

**Figure 3.**
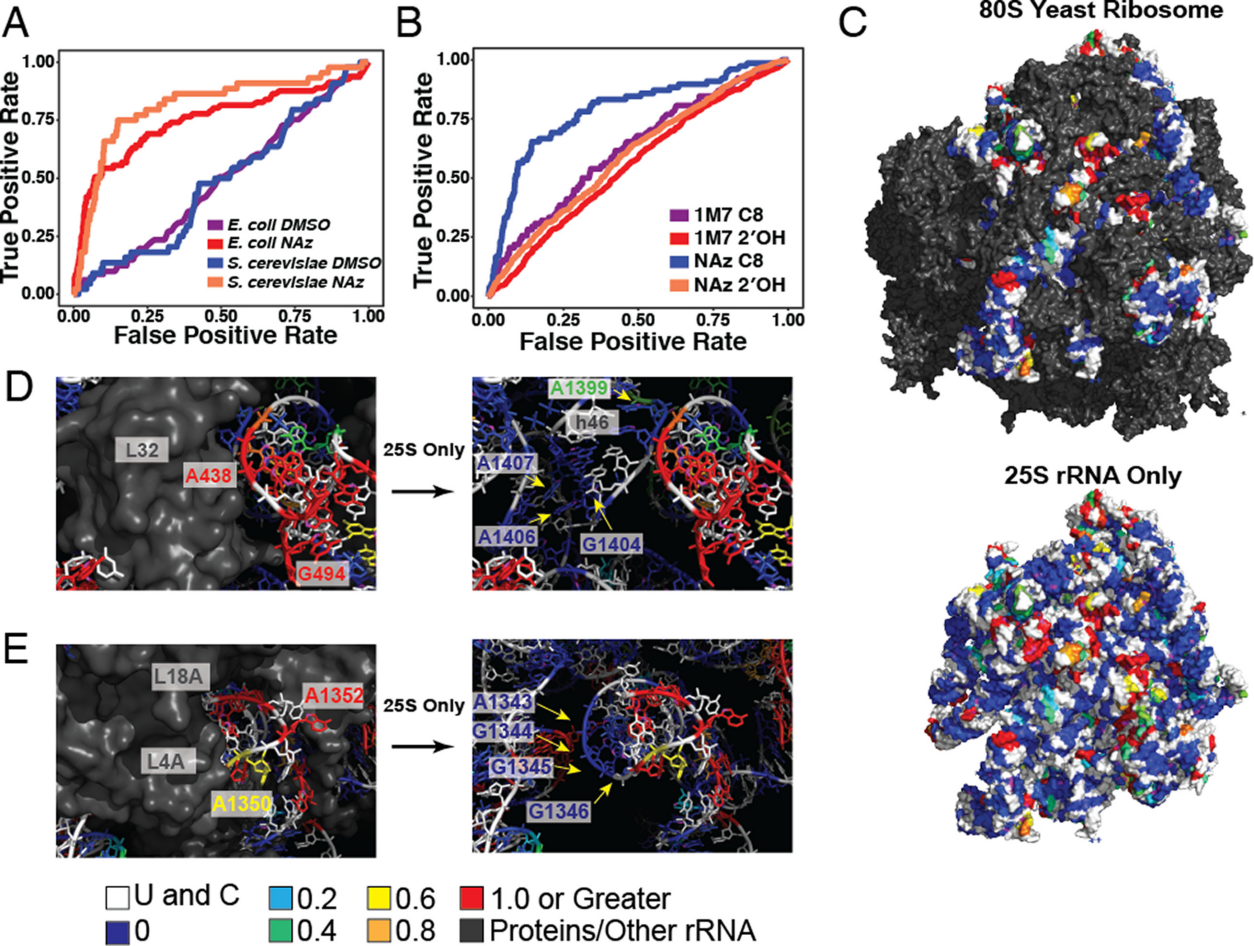
NAz reactivity is an effective predictor of solvent accessibility. (**A**) ROC curves for prediction of solvent-accessible nucleotides from LASER-MaP on purified ribosomes from *E. coli* and *S. cerevisiae*. SASA was calculated with a probe radius of 4 Å and true positives were defined as residues that had C8 solvent accessibility greater than or equal to 5 Å^2^. All other nucleotides are considered true negatives. (**B**) ROC curves comparing SHAPE-MaP (1M7) with LASER-MaP (NAz), demonstrating that LASER-MaP measures solvent accessibility while SHAPE-MaP does not. (**C**) NAz reactivity overlaid onto the 25s rRNA in complex with the rest of *S. cerevisiae* ribosome or alone. Structure from (46) (PDB ID 4V88). The ribosome is oriented with the small subunit to the left, the A site at the bottom, and the E site on top. All rRNA and protein except for the 25S rRNA are colored in gray. Color scale for NAz reactivity, in percent mutation, is shown under panel E. (**D, E**) Panels showing specific areas of NAz reactivity on the *S. cerevisiae* X-Ray structure.

To test the utility of LASER-MaP as a predictor of solvent accessibility, we compared the mutation rate at each A or G nucleotide in the *E. coli* ribosome with the computed solvent-accessible surface area (SASA) of the C8 atom for the same nucleotide, based on a high resolution X-ray crystal structure (45). We computed SASA with NAz approximated as a sphere with a 4 Å radius and defined solvent-accessible (true positive) nucleotides as purines with a SASA of 5 Å^2^ or more. We generated receiver operating characteristic (ROC) curves (Figure 3A) that test how well the LASER-MaP signal can separate true positives from false positives (nucleotides with SASA <5 Å^2^) at different thresholds of mutation rate. The area under the ROC curve (AUC) quantifies the predictive value of the measurement, with 1.0 indicating the existence of a mutation rate threshold that detects all true positives with no false positives, and 0.50 indicating no predictive value above random chance. NAz reactivity was a good predictor of solvent accessibility (AUC = 0.75) compared to our DMSO control (AUC = 0.49). Our reported AUC is lower than the value found in a similar experiment performed on yeast ribosomes with DMS (14); however, for DMS the true positives/negatives can be defined by the base-pairing status of the nucleotide as well as SASA, while we are using SASA alone, which requires arbitrary choices of cutoff and solvent radius for its computation. The ROC curves were robust to the choice of probe radius or SASA cutoff. Slight increases in sensitivities were observed as the SASA cutoff for true positives was increased (Supplementary Figure S6A–D), and we only observed slight variations at probe radii >4 Å (Supplementary Figure S6A–D). To further test our approach, we compared our LASER-MaP data for *S. cerevisiae* ribosomes to the *S. cerevisiae* ribosome crystal structure (46). Our ROC curves indicate a similar trend as seen for *E. coli* ribosomes, with an AUC of 0.82 (Figure 3A). Broadly speaking, these results demonstrate that LASER is an accurate tool for measuring solvent accessibility.

Differences in nucleotide reactivity between reagents can be used to predict more accurate structures of a given RNA (47). Using *S. cerevisiae* ribosomes as our model (46), we calculated the SASA of 2′OH and generated ROC curves with the LASER-MaP and SHAPE-MaP (1M7) mutation frequencies (Figure 3B). As expected, SHAPE reactivities are poor predictors of the solvent accessibility of 2′OH positions (AUC = 0.56). LASER-MaP accurately detects the solvent accessibility of C8 positions (AUC = 0.81) but not of the 2′OH in the same nucleotide (AUC = 0.59). Unexpectedly, SHAPE reactivity was weakly predictive of C8 accessibility (Figure 3B). We reason that this is due to a correlation of positional flexibility with C8 accessibility, leading to an increase in modification of the 2′OH. These results show that LASER provides structural information complementary to that provided by SHAPE.

In the course of our analysis, we found the computation of C8 SASA to be relatively crude, yielding few C8 atoms with measurable SASA, even in exposed regions of the crystal structures. To further examine NAz reactivity in solvent exposed regions, we superimposed LASER-MaP reactivity onto the X-Ray structure of the *S. cerevisiae* ribosome (Figure 3C) (46). Upon inspection, the majority of unreactive residues appeared protected from solvent, and exposed regions of the 25S rRNA displayed various degrees of protections. For example, residues 1395–1414 of helix 46 display low solvent accessibility due to protection by ribosomal protein L32 (Figure 3D). Nucleotides from the loop (G1404, A1406, A1407) contact L32, and the rest of helix 46 is buried inside the complex. Slight reactivity was observed for residue A1399 which is deeper in a ribosome pocket but has its C8 atom exposed to solvent. Adjacent residues A438 and G494 are not covered by either rRNA or proteins and had high NAz reactivity. Similar protections occurred around helix 45 (Figure 3E), whose loop (G1349, A1350, A1352, G1354, A1355) is fully exposed and whose stem (A1343–G1346) is buried within the ribosome with C8 atoms pointing towards ribosomal proteins L4A and L18A.

We also examined regions with no computed solvent accessibility, but high NAz reactivity, some of which are depicted in Supplementary Figure S6. In each of these cases, such as G763 and A2222, manual inspection revealed C8 positions that were exposed to solvent with the residues not base paired in the crystal structure. The high mutation rate at these positions indicates that there could be multiple conformations in solution susceptible to NAz modifications. SASA computation uses one conformation of the structure and could overlook residues with multiple conformations in solution. As such, these discrepancies could be due to a combination of studying the static structure and crude SASA modeling, but with a local structure highly open and reactive to NAz in solution. These observations further support the notion that NAz is reacting with solvent exposed residues.

### LASER-MaP can be used to monitor binding of ligands to ribosomal RNA

Differential chemical probing analysis is a powerful technique for identifying conformational changes, as well as the binding sites of proteins and small molecules in RNA complexes such as the ribosome (4,5,48). Upon ligand binding, nucleotides in the vicinity of the binding site are ‘protected’, becoming less accessible and thus unreactive to covalent modifying agents. Secondary protections and deprotections, further away from the binding site, may be indicative of larger conformational changes in the RNA induced by ligand binding. Probing with reagents such as DMS, kethoxal and CMCT has yielded enormous insight into ribosome structure and function (4,48–50), but has been limited by the small number of unpaired nucleotides in the rRNA that are reactive to these agents, as well as the large number of primer extension gels required to survey such a large structure (48). This second bottleneck has recently been resolved by high-throughput sequencing methodologies (10–12,14–17,19,20,25), but the underlying issue of probe reactivity persists.

**Figure 4.**
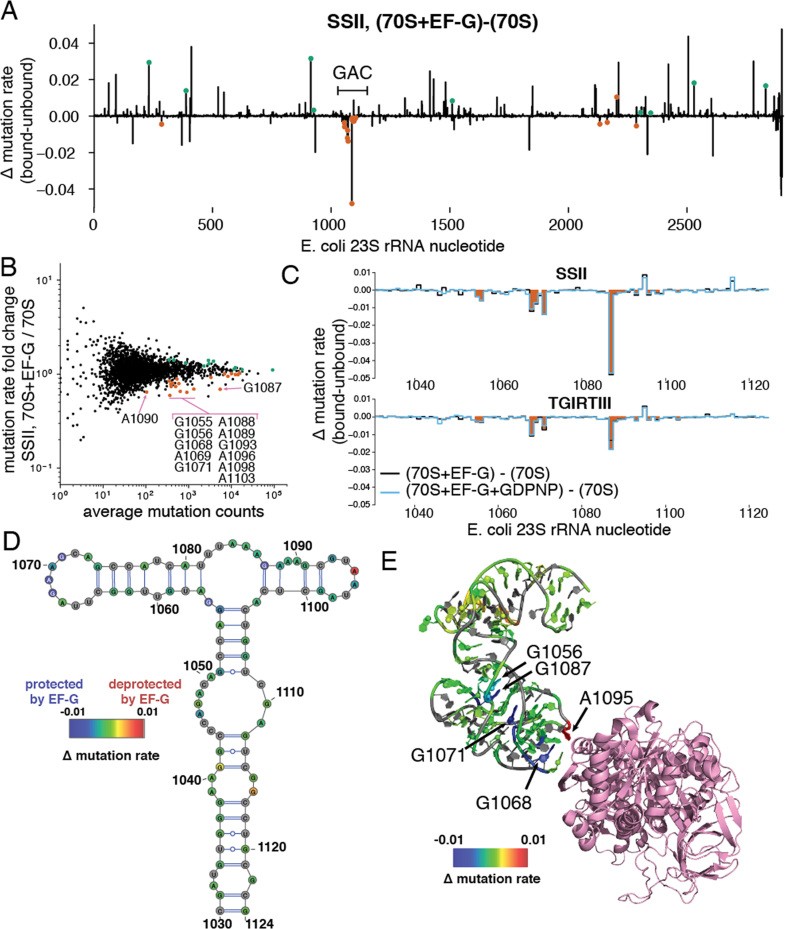
Detection of elongation factor G binding to the GTPase activation center of the *E. coli* ribosome using LASER-MaP. (**A**) Difference in LASER-MaP mutation fraction across the entire *E. coli* 23S rRNA, for EF-G bound ribosomes compared to unbound ribosomes. The location of the GTPase activating center (GAC) is indicated. Nucleotides with a statistically significant increase or decrease in LASER-MaP signal among all EF-G-treated samples are highlighted in green and orange, respectively. The nucleotides that show significant protections but have a positive change in mutation rate are artifacts of rescaling mutation rates for calling hits (See methods), which was not done in these figures. (**B**) MA (log average vs log ratio) plot comparing the average number of mutations at rRNA nucleotides to their fold change in mutation fraction upon EF-G binding. Nucleotides with a statistically significant increase or decrease in all EF-G treated samples are highlighted in green and orange, respectively. Protected nucleotides in the GAC are labeled. (**C**) Detail of panel A limited to the GAC. Protected nucleotides are shaded in orange. (**D**) Differences in LASER-MaP mutation fraction (SSII, EF-G bound minus unbound ribosomes) overlaid on the secondary structure of the GTPase activation center of the *E. coli* 23S rRNA. Pyrimidine nucleotides are colored grey. Figure generated using VARNA (http://varna.lri.fr/) (59). (**E**) Differences in LASER-MaP mutation fraction (SSII, EF-G bound minus unbound ribosomes) overlaid on the GTPase activation center of the EF-G-bound *E. coli* ribosome (PDB ID 3J9Z), viewed from the A site side of the ribosome. C8 atoms of purines in the GAC are shown as spheres, EF-G is shown in pink cartoon diagram, and pyrimidines are gray. Coloring of RNA by difference values was performed with RiboVis (https://ribokit.github.io/RiboVis/). Arrows highlight a subset of EF-G protected nucleotides, as well as A1095 which shows increased reactivity that was not determined to be statistically significant.

To test the utility of LASER-Seq for the identification of ligand binding sites, we performed LASER-MaP on purified *E. coli* ribosomes incubated with elongation factor G (EF-G), with or without the non-hydrolyzable GTP analog GDPNP. GDPNP is expected to lock EF-G in a ribosome-bound state (51) and thus increase the likelihood of observing protections. We did not see major differences in mutation rate in the presence or absence of GDPNP, so we treated these samples as replicates. To identify sites of altered NAz reactivity upon EF-G binding, we adapted a Poisson counting error model that was previously used for differential SHAPE-MaP analysis (38). In this analysis, larger absolute numbers of mutations and absolute differences in mutation rate are more likely to be called real changes. A number of nucleotides were reproducibly protected or deprotected in the EF-G bound samples (Figure 4A, Supplementary Figure S7A, Supplementary Table S2) with both RT enzymes. We also produced MA plots comparing the fold change in mutation for each nucleotide upon EF-G incubation to its average number of mutations between both datasets (Figure 4B, Supplementary Figure S7B). These plots show that many of the statistically-detected reactivity changes were in nucleotides with large numbers of mutations, but with small fold changes within the spread observed for other nucleotides with similar mutation rates. This suggests that these statistical calls are spurious.

The remaining protected nucleotides cluster in the GTPase-activation center (GAC) of the 23S rRNA, immediately adjacent to the known EF-G binding site (Figure 4C, D and E) (52). Crystal structures of the *E. coli* ribosome alone or bound to EF-G (45,52) show this entire region of the GAC moving upon EF-G binding (Supplementary Figure S7C), consistent with the large number of protections in this region. The protections further from the contact site with EF-G could indicate compression of the GAC RNA upon EF-G binding, which might limit the ability of NAz to access C8 atoms therein. The interaction of EF-G with the ribosome was previously analyzed by probing with DMS and primer extension (53), but only two protections (A1067, A1069) were identified in this region. This is probably due to the paucity of unpaired DMS-reactive nucleotides in this region, as well as the reduced sensitivity of gel-based RT stop measurement. LASER-MaP, however, readily identified EF-G induced conformational changes at both paired and unpaired nucleotides in this region. These results demonstrate the utility of LASER-MaP as a tool to interrogate protein binding to large and base-paired RNAs.

**Figure 5.**
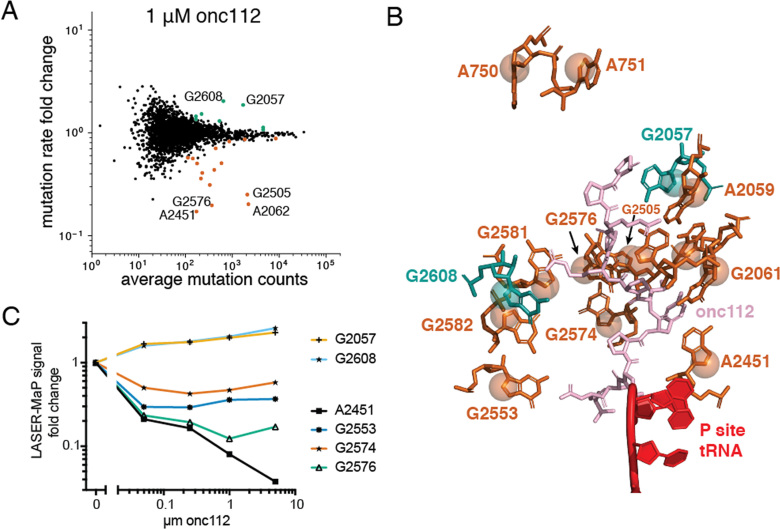
Detection of onc112 binding to the *E. coli* ribosome using LASER-MaP. (**A**) MA plot comparing the average number of mutations at rRNA nucleotides to their fold change in mutation fraction upon onc112 incubation. Nucleotides with a statistically significant increase or decrease in all treated samples are highlighted in green and orange, respectively. Select nucleotides are labeled. (**B**) View of the peptidyl transferase center and peptide tunnel of the *T. Thermophilus* ribosome bound to onc112 from PDB ID 4Z8C. Nucleotides homologous to protected or deprotected nucleotides were identified by sequence alignment and highlighted in orange and green, respectively. C8 atoms are shown as spheres. (**C**) Onc112-dependent changes in LASER-MaP signal for select nucleotides. UV-only background was subtracted from all data points before dividing each by the average of the 0μM onc112 replicates.

In order to more directly test the reproducibility and quantitative nature of LASER-MaP, we performed an additional batch of *in vitro* probing experiments with *E. coli* ribosomes. We incubated 1μM ribosomes with several concentrations of the proline-rich antimicrobial peptide onc112 (54–56) and performed LASER-MaP with SSII. We found that the reproducibility within a batch of samples was higher than between batches (Supplementary Figure S8A). This difference disappeared upon background subtraction. Using the same analytical method as for EF-G, we identified a number of rRNA residues which were protected or deprotected by onc112 binding (Supplementary Figures 5A and S8B. We superimposed these nucleotide positions onto the previously-determined structure of the *Thermus thermophilus* 70S ribosome co-crystallized with onc112 (56) (Supplementary Table S3). These positions cluster around the binding site of onc112 in the peptide exit tunnel (Figure 5B). The ribosome concentration used in this experiment was too high for accurate measurement of binding constants, but many nucleotides displayed monotonic increases or decreases in LASER-MaP signal with onc112 concentration (Figure 5C), indicating that LASER-MaP can provide a semi-quantitative if not fully quantitative measure of ligand binding. Other nucleotides behaved in more complex ways, with the signal plateauing or changing direction at high onc112 concentrations. This could mean, among other explanations, that these nucleotides have different dynamics for onc112 binding, or that background noise is dominating the reduced mutational signal at high onc112 concentrations.

## DISCUSSION

The recent advent of high-throughput RNA structure analysis methods has greatly advanced our ability to analyze the structures of transcriptomes and large RNA molecules. Here we expand the existing structure probing toolbox by adapting LASER into LASER-Seq and LASER-MaP. LASER has reactivity preferences that depend on solvent accessibility, making it orthogonal to other probing methods that depend on RNA base-pairing. We have thoroughly characterized these methods with ribosomes from 3 different species, both *in vitro* and *in vivo*, to show that they produce RT stops or nucleotide mutations that agree with solvent accessibilities computed from high-resolution crystal or cryo-EM structures, as well as manual gel-based LASER analysis.

LASER-MaP is sensitive and able to modify base-paired nucleotides, making it well suited to detecting ligand binding sites on RNAs. We demonstrated this by recapitulating the binding sites of EF-G and onc112 on *E. coli* ribosomes and identifying more protections in the same rRNA region than were detected by older methods. Our results are suggestive of a large-scale movement and compression of the GAC caused by EF-G binding, which is supported by structures of EF-G bound ribosomes, while the previous result could only detect the proximal binding site of EF-G. These distal protections raise the possibility that NAz reactivity is affected by the spacing or curvature of RNA helices, a facet of NAz probing that requires further analysis. If true, this effect could be useful for determining additional constraints on unknown RNA structures. The ability to detect translation-factor and small molecule binding means that LASER-MaP could aid in determining the mechanism of action of ribosome-targeting antibiotics *in vitro* or *in vivo*, by monitoring the conformational state of the ribosome after treatment with a drug (44,49,57), while simultaneously detecting the direct binding site.

LASER-Seq and LASER-MaP can be readily adapted to the transcriptome-wide probing of mRNA structures with the addition of rRNA depletion or poly-A selection. This will enable more precise predictions of mRNA structure in combination with existing SHAPE- and DMS-based approaches and may be able to provide information on protein binding sites in RNA that are not detectable by other methods. Our pilot experiments indicate that mutations occur at NAz-reactive nucleotides at a frequency greater than SHAPE reagents, and comparable to DMS for G nucleotides, so sequencing coverage requirements should be no higher than for other MaP techniques. Recent analyses of DMS probing data suggest that RT-stops and mutations provide complementary information, and both may be needed to provide a complete picture of the state of chemical modification (58) (BioRxiv: https://doi.org/10.1101/292532, https://doi.org/10.1101/176883). More work is required to determine if this is true for LASER, and to integrate RT-stop and MaP data from complementary probes into a single analytical framework for RNA structure prediction. We envision that LASER-Seq and LASER-MaP will be immediately applicable to many existing problems, from the identification of protein and small-molecule binding sites in large RNAs, to transcriptome-wide prediction of RNA structure and solvent accessibility.

## DATA AVAILABILITY

The data analysis pipeline and outputs tables of RT stops and mutations is available on GitHub (https://github.com/borisz264/LASER_seq_2018). Raw reads and processed data have been deposited in the Gene Expression Omnibus with accession GSE113529.

## Supplementary Material

Supplementary Data

## References

[1] Wan Y., Kertesz M., Spitale R.C., Segal E., Chang H.Y. Understanding the transcriptome through RNA structure. Nat. Rev. Genet. 2011; 12:641–655. 21850044 10.1038/nrg3049 PMC3858389

[2] Mortimer S.A., Kidwell M.A., Doudna J.A. Insights into RNA structure and function from genome-wide studies. Nat. Rev. Genet. 2014; 15:469–479. 24821474 10.1038/nrg3681

[3] Merino E.J., Wilkinson K.A., Coughlan J.L., Weeks K.M. RNA structure analysis at single nucleotide resolution by selective 2′-hydroxyl acylation and primer extension (SHAPE). J. Am. Chem. Soc. 2005; 127:4223–4231. 15783204 10.1021/ja043822v

[4] Tijerina P., Mohr S., Russell R. Dms footprinting of structured rnas and rna-protein complexes. Nat. Protoc. 2007; 2:2608–2623. 17948004 10.1038/nprot.2007.380 PMC2701642

[5] Inoue T., Cech T.R. Secondary structure of the circular form of the Tetrahymena rRNA intervening sequence: a technique for RNA structure analysis using chemical probes and reverse transcriptase. Proc. Natl. Acad. Sci. U.S.A. 1985; 82:648–652. 2579378 10.1073/pnas.82.3.648 PMC397102

[6] Powers T., Noller H.F. Hydroxyl radical footprinting of ribosomal proteins on 16S rRNA. RNA. 1995; 1:194–209. 7585249 PMC1369073

[7] Hulscher R.M., Bohon J., Rappé M.C., Gupta S., D’Mello R., Sullivan M., Ralston C.Y., Chance M.R., Woodson S.A. Probing the structure of ribosome assembly intermediates in vivo using DMS and hydroxyl radical footprinting. Methods. 2016; 103:49–56. 27016143 10.1016/j.ymeth.2016.03.012 PMC4921310

[8] Feng C., Chan D., Joseph J., Muuronen M., Coldren W.H., Dai N., Corrêa I.R., Furche F., Hadad C.M., Spitale R.C. Light-activated chemical probing of nucleobase solvent accessibility inside cells. Nat. Chem. Biol. 2018; 14:276–283. 29334380 10.1038/nchembio.2548 PMC6203945

[9] Ritchey L.E., Su Z., Tang Y., Tack D.C., Assmann S.M., Bevilacqua P.C. Structure-seq2: sensitive and accurate genome-wide profiling of RNA structure in vivo. Nucleic Acids Res. 2017; 45:e135. 28637286 10.1093/nar/gkx533 PMC5737731

[10] Ding Y., Tang Y., Kwok C.K., Zhang Y., Bevilacqua P.C., Assmann S.M. In vivo genome-wide profiling of RNA secondary structure reveals novel regulatory features. Nature. 2014; 505:696–700. 24270811 10.1038/nature12756

[11] Lovejoy A.F., Riordan D.P., Brown P.O. Transcriptome-Wide mapping of Pseudouridines: Pseudouridine synthases modify specific mRNAs in S. cerevisiae. PLoS One. 2014; 9:e110799. 25353621 10.1371/journal.pone.0110799 PMC4212993

[12] Lucks J.B., Mortimer S.A., Trapnell C., Luo S., Aviran S., Schroth G.P., Pachter L., Doudna J.A., Arkin A.P. Multiplexed RNA structure characterization with selective 2′-hydroxyl acylation analyzed by primer extension sequencing (SHAPE-Seq). Proc. Natl. Acad. Sci. U.S.A. 2011; 108:11063–11068. 21642531 10.1073/pnas.1106501108 PMC3131332

[13] Spitale R.C., Flynn R.A., Zhang Q.C., Crisalli P., Lee B., Jung J.-W., Kuchelmeister H.Y., Batista P.J., Torre E.A., Kool E.T. Structural imprints in vivo decode RNA regulatory mechanisms. Nature. 2015; 519:486–490. 25799993 10.1038/nature14263 PMC4376618

[14] Rouskin S., Zubradt M., Washietl S., Kellis M., Weissman J.S. Genome-wide probing of RNA structure reveals active unfolding of mRNA structures in vivo. Nature. 2014; 505:701–705. 24336214 10.1038/nature12894 PMC3966492

[15] Hector R.D., Burlacu E., Aitken S., Le Bihan T., Tuijtel M., Zaplatina A., Cook A.G., Granneman S. Snapshots of pre-rRNA structural flexibility reveal eukaryotic 40S assembly dynamics at nucleotide resolution. Nucleic Acids Res. 2014; 42:12138–12154. 25200078 10.1093/nar/gku815 PMC4231735

[16] Talkish J., May G., Lin Y., Woolford J.L., McManus C.J., McManus C.J. Mod-seq: high-throughput sequencing for chemical probing of RNA structure. RNA. 2014; 20:713–720. 24664469 10.1261/rna.042218.113 PMC3988572

[17] Incarnato D., Neri F., Anselmi F., Oliviero S. Genome-wide profiling of mouse RNA secondary structures reveals key features of the mammalian transcriptome. Genome Biol. 2014; 15:491. 25323333 10.1186/s13059-014-0491-2 PMC4220049

[18] Carlile T.M., Rojas-Duran M.F., Zinshteyn B., Shin H., Bartoli K.M., Gilbert W.V. Pseudouridine profiling reveals regulated mRNA pseudouridylation in yeast and human cells. Nature. 2014; 515:143–146. 25192136 10.1038/nature13802 PMC4224642

[19] Schwartz S., Bernstein D.A., Mumbach M.R., Jovanovic M., Herbst R.H., León-Ricardo B.X., Engreitz J.M., Guttman M., Satija R., Lander E.S. Transcriptome-wide mapping reveals widespread dynamic-regulated pseudouridylation of ncRNA and mRNA. Cell. 2014; 159:148–162. 25219674 10.1016/j.cell.2014.08.028 PMC4180118

[20] Kielpinski L.J., Vinther J. Massive parallel-sequencing-based hydroxyl radical probing of RNA accessibility. Nucleic Acids Res. 2014; 42:e70. 24569351 10.1093/nar/gku167 PMC4005689

[21] Schuller A.P., Zinshteyn B., Enam S.U., Green R. Directed hydroxyl radical probing reveals Upf1 binding to the 80S ribosomal E site rRNA at the L1 stalk. Nucleic Acids Res. 2018; 46:2060–2073. 29253221 10.1093/nar/gkx1263 PMC5829565

[22] Siegfried N.A., Busan S., Rice G.M., Nelson J.A.E., Weeks K.M. RNA motif discovery by SHAPE and mutational profiling (SHAPE-MaP). Nat. Methods. 2014; 11:959–965. 25028896 10.1038/nmeth.3029 PMC4259394

[23] Zubradt M., Gupta P., Persad S., Lambowitz A.M., Weissman J.S., Rouskin S. DMS-MaPseq for genome-wide or targeted RNA structure probing in vivo. Nat. Methods. 2016; 14:75–82. 27819661 10.1038/nmeth.4057 PMC5508988

[24] Homan P.J., Favorov O.V., Lavender C.A., Kursun O., Ge X., Busan S., Dokholyan N.V., Weeks K.M. Single-molecule correlated chemical probing of RNA. Proc. Natl. Acad. Sci. U.S.A. 2014; 111:13858–13863. 25205807 10.1073/pnas.1407306111 PMC4183288

[25] Carlile T.M., Rojas-Duran M.F., Zinshteyn B., Shin H., Bartoli K.M., Gilbert W.V. Pseudouridine profiling reveals regulated mRNA pseudouridylation in yeast and human cells. Nature. 2014; 515:143–146. 25192136 10.1038/nature13802 PMC4224642

[26] Mortimer S.A., Trapnell C., Aviran S., Pachter L., Lucks J.B. SHAPE-Seq: high-throughput RNA structure analysis. Curr. Protoc. Chem. Biol. 2012; 4:275–297. 23788555 10.1002/9780470559277.ch120019

[27] Shaw J.J., Green R. Two distinct components of release factor function uncovered by nucleophile partitioning analysis. Mol. Cell. 2007; 28:458–467. 17996709 10.1016/j.molcel.2007.09.007 PMC2175178

[28] Blanchard S.C., Kim H.D., Gonzalez R.L. Jr, Puglisi J.D., Chu S. tRNA dynamics on the ribosome during translation. Proc. Natl. Acad. Sci. U.S.A. 2004; 101:12893–12898. 15317937 10.1073/pnas.0403884101 PMC516491

[29] Eyler D.E., Green R. Distinct response of yeast ribosomes to a miscoding event during translation. RNA. 2011; 17:925–932. 21415142 10.1261/rna.2623711 PMC3078741

[30] Rio D.C., Ares M.J., Hannon G.J., Nilsen T.W. RNA: a laboratory manual. 2011; Cold Spring Harbor Laboratory Press.

[31] McGlincy N.J., Ingolia N.T. Transcriptome-wide measurement of translation by ribosome profiling. Methods. 2017; 126:112–129. 28579404 10.1016/j.ymeth.2017.05.028 PMC5582988

[32] Jiang H., Lei R., Ding S.-W., Zhu S. Skewer: a fast and accurate adapter trimmer for next-generation sequencing paired-end reads. BMC Bioinformatics. 2014; 15:182. 24925680 10.1186/1471-2105-15-182 PMC4074385

[33] Busan S., Weeks K.M. Accurate detection of chemical modifications in RNA by mutational profiling (MaP) with ShapeMapper 2. RNA. 2018; 24:143–148. 29114018 10.1261/rna.061945.117 PMC5769742

[34] Kurylo C.M., Alexander N., Dass R.A., Parks M.M., Altman R.A., Vincent C.T., Mason C.E., Blanchard S.C. Genome sequence and analysis of Escherichia coli MRE600, a colicinogenic, nonmotile strain that lacks RNase I and the Type I methyltransferase, EcoKI. Genome Biol. Evol. 2016; 8:742–752. 26802429 10.1093/gbe/evw008 PMC4825418

[35] Homann O.R., Johnson A.D. MochiView: versatile software for genome browsing and DNA motif analysis. BMC Biol. 2010; 8:49. 20409324 10.1186/1741-7007-8-49 PMC2867778

[36] Robinson J.T., Thorvaldsdóttir H., Winckler W., Guttman M., Lander E.S., Getz G., Mesirov J.P. Integrative Genomics Viewer. Nat. Biotechnol. 2011; 29:24–26. 21221095 10.1038/nbt.1754 PMC3346182

[37] Dobin A., Davis C.A., Schlesinger F., Drenkow J., Zaleski C., Jha S., Batut P., Chaisson M., Gingeras T.R. STAR: ultrafast universal RNA-seq aligner. Bioinformatics. 2013; 29:15–21. 23104886 10.1093/bioinformatics/bts635 PMC3530905

[38] Smola M.J., Calabrese J.M., Weeks K.M. Detection of RNA–Protein interactions in living cells with SHAPE. Biochemistry. 2015; 54:6867–6875. 26544910 10.1021/acs.biochem.5b00977 PMC4900165

[39] Moazed D., Noller H.F. Transfer RNA shields specific nucleotides in 16S ribosomal RNA from attack by chemical probes. Cell. 1986; 47:985–994. 2430725 10.1016/0092-8674(86)90813-5

[40] Lecanda A., Nilges B.S., Sharma P., Nedialkova D.D., Schwarz J., Vaquerizas J.M., Leidel S.A. Dual randomization of oligonucleotides to reduce the bias in ribosome-profiling libraries. Methods. 2016; 107:89–97. 27450428 10.1016/j.ymeth.2016.07.011 PMC5024760

[41] Weinberg D.E., Shah P., Eichhorn S.W., Hussmann J.A., Plotkin J.B., Bartel D.P. Improved Ribosome-Footprint and mRNA measurements provide insights into dynamics and regulation of yeast translation. Cell Rep. 2016; 14:1787–1799. 26876183 10.1016/j.celrep.2016.01.043 PMC4767672

[42] Tunney R., McGlincy N.J., Graham M.E., Naddaf N., Pachter L., Lareau L.F. Accurate design of translational output by a neural network model of ribosome distribution. Nat. Struct. Mol. Biol. 2018; 25:1–6. 29967537 10.1038/s41594-018-0080-2 PMC6457438

[43] Weeks K.M., Mauger D.M. Exploring RNA structural codes with SHAPE chemistry. Acc. Chem. Res. 2011; 44:1280–1291. 21615079 10.1021/ar200051h PMC3177967

[44] McClary B., Zinshteyn B., Meyer M., Jouanneau M., Pellegrino S., Yusupova G., Schuller A., Reyes J.C.P., Lu J., Guo Z. Inhibition of eukaryotic translation by the antitumor natural product agelastatin A. Cell Chem. Biol. 2017; 24:605–613. 28457705 10.1016/j.chembiol.2017.04.006 PMC5562292

[45] Noeske J., Wasserman M.R., Terry D.S., Altman R.B., Blanchard S.C., Cate J.H.D. High-resolution structure of the Escherichia coli ribosome. Nat. Struct. Mol. Biol. 2015; 22:336–341. 25775265 10.1038/nsmb.2994 PMC4429131

[46] Ben-Shem A., Garreau de Loubresse N., Melnikov S., Jenner L., Yusupova G., Yusupov M. The structure of the eukaryotic ribosome at 3.0 A resolution. Science. 2011; 334:1524–1529. 22096102 10.1126/science.1212642

[47] Rice G.M., Leonard C.W., Weeks K.M. RNA secondary structure modeling at consistent high accuracy using differential SHAPE. RNA. 2014; 20:846–854. 24742934 10.1261/rna.043323.113 PMC4024639

[48] Moazed D., Stern S., Noller H.F. Rapid chemical probing of conformation in 16 S ribosomal RNA and 30 S ribosomal subunits using primer extension. J. Mol. Biol. 1986; 187:399–416. 2422386 10.1016/0022-2836(86)90441-9

[49] Moazed D., Noller H.F. Interaction of antibiotics with functional sites in 16S ribosomal RNA. Nature. 1987; 327:389–394. 2953976 10.1038/327389a0

[50] Hogan J.J., Gutell R.R., Noller H.F. Probing the conformation of 26S rRNA in yeast 60S ribosomal subunits with kethoxal. Biochemistry. 1984; 23:3330–3335. 6380585 10.1021/bi00309a033

[51] Kuriki Y., Inoue N., Kaziro Y. Formation of a complex between GTP, G factor, and ribosomes as an intermediate of ribosome-dependent GTPase reaction. Biochim. Biophys. Acta - Nucleic Acids Protein Synth. 1970; 224:487–497. 10.1016/0005-2787(70)90581-2 5498080

[52] Li W., Liu Z., Koripella R.K., Langlois R., Sanyal S., Frank J. Activation of GTP hydrolysis in mRNA-tRNA translocation by elongation factor G. Sci. Adv. 2015; 1:e1500169. 26229983 10.1126/sciadv.1500169 PMC4517844

[53] Moazed D., Robertson J.M., Noller H.F. Interaction of elongation factors EF-G and EF-Tu with a conserved loop in 23S RNA. Nature. 1988; 334:362–364. 2455872 10.1038/334362a0

[54] Seefeldt A.C., Nguyen F., Antunes S., Pérébaskine N., Graf M., Arenz S., Inampudi K.K., Douat C., Guichard G., Wilson D.N. The proline-rich antimicrobial peptide Onc112 inhibits translation by blocking and destabilizing the initiation complex. Nat. Struct. Mol. Biol. 2015; 22:470–475. 25984971 10.1038/nsmb.3034

[55] Roy R.N., Lomakin I.B., Gagnon M.G., Steitz T.A. The mechanism of inhibition of protein synthesis by the proline-rich peptide oncocin. Nat. Struct. Mol. Biol. 2015; 22:466–469. 25984972 10.1038/nsmb.3031 PMC4456192

[56] Gagnon M.G., Roy R.N., Lomakin I.B., Florin T., Mankin A.S., Steitz T.A. Structures of proline-rich peptides bound to the ribosome reveal a common mechanism of protein synthesis inhibition. Nucleic Acids Res. 2016; 44:2439–2450. 26809677 10.1093/nar/gkw018 PMC4797290

[57] Schneider-Poetsch T., Ju J., Eyler D.E., Dang Y., Bhat S., Merrick W.C., Green R., Shen B., Liu J.O. Inhibition of eukaryotic translation elongation by cycloheximide and lactimidomycin. Nat. Chem. Biol. 2010; 6:209–217. 20118940 10.1038/nchembio.304 PMC2831214

[58] Sexton A.N., Wang P.Y., Rutenberg-Schoenberg M., Simon M.D. Interpreting reverse transcriptase termination and mutation events for greater insight into the chemical probing of RNA. Biochemistry. 2017; 56:4713–4721. 28820243 10.1021/acs.biochem.7b00323 PMC5648349

[59] Darty K., Denise A., Ponty Y. VARNA: interactive drawing and editing of the RNA secondary structure. Bioinformatics. 2009; 25:1974–1975. 19398448 10.1093/bioinformatics/btp250 PMC2712331

