# Supplementary material for: Assaying RNA structure with LASER-Seq
Source: Nucleic Acids Res. 2018 Nov 22;47(1):43–55. doi: 10.1093/nar/gky1172 (PMC6326810; doi:10.1093/nar/gky1172)
Supplement: Supplementary Data [file gky1172_supplemental_files.zip › 180831_LASER_SEQ_SuppInfo_.docx]

**Assaying­­­­ RNA Structure with LASER-Seq**

Boris Zinshteyn*^1^, Dalen Chan^2^, Whitney England^2^, Chao Feng^2^, Rachel Green^1,3^, Robert C. Spitale*^2,3^

(1) Department of Molecular Biology and Genetics. Johns Hopkins University. Baltimore, Maryland 21205 (2) Department of Pharmaceutical Sciences, (3) Howard Hughes Medical Institute, Johns Hopkins University School of Medicine, Baltimore, Maryland 21205, (4) Department of Chemistry. University of California, Irvine. Irvine, California. 92697

Correspondence:

[rspitale@uci.edu](mailto:rspitale@uci.edu)

borisz@jhmi.edu

**
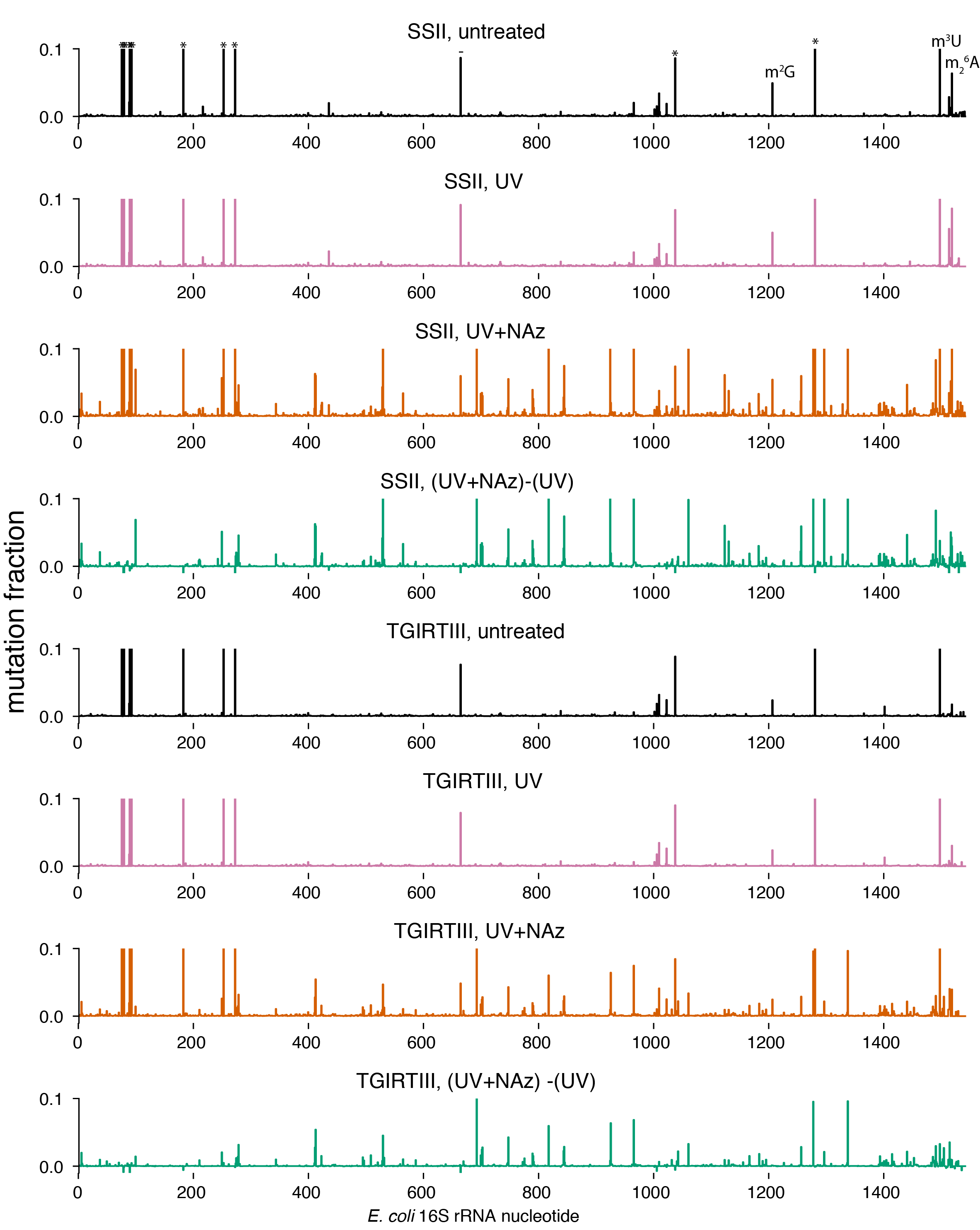
**

**Figure S1: LASER-MaP reactivity for the entire *E. coli* 16S rRNA**

Mutation fractions for SSII and TGIRTIII. Single nucleotide polymorphisms between *E. coli* rRNA copies are indicated by *, deletions by -, and modified nucleotides that cause mutations or RT stops are indicated by name.


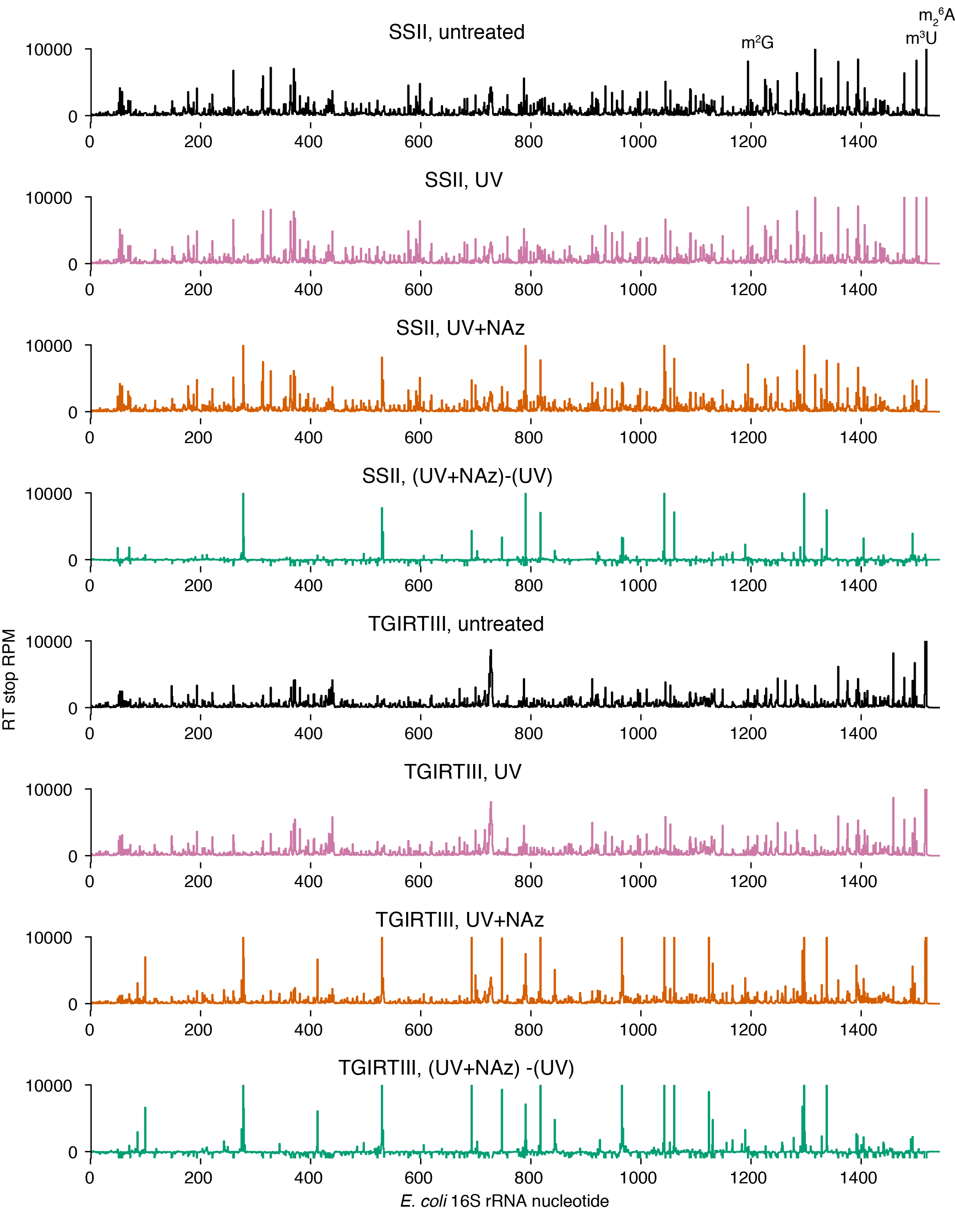


**Figure S2: LASER-Seq reactivity for the entire *E. coli* 16S rRNA**

RT stop RPMs for SSII and TGIRTIII. Single nucleotide polymorphisms between *E. coli* rRNA copies are indicated by *, deletions by -, and modified nucleotides that cause mutations or RT stops are indicated by name.


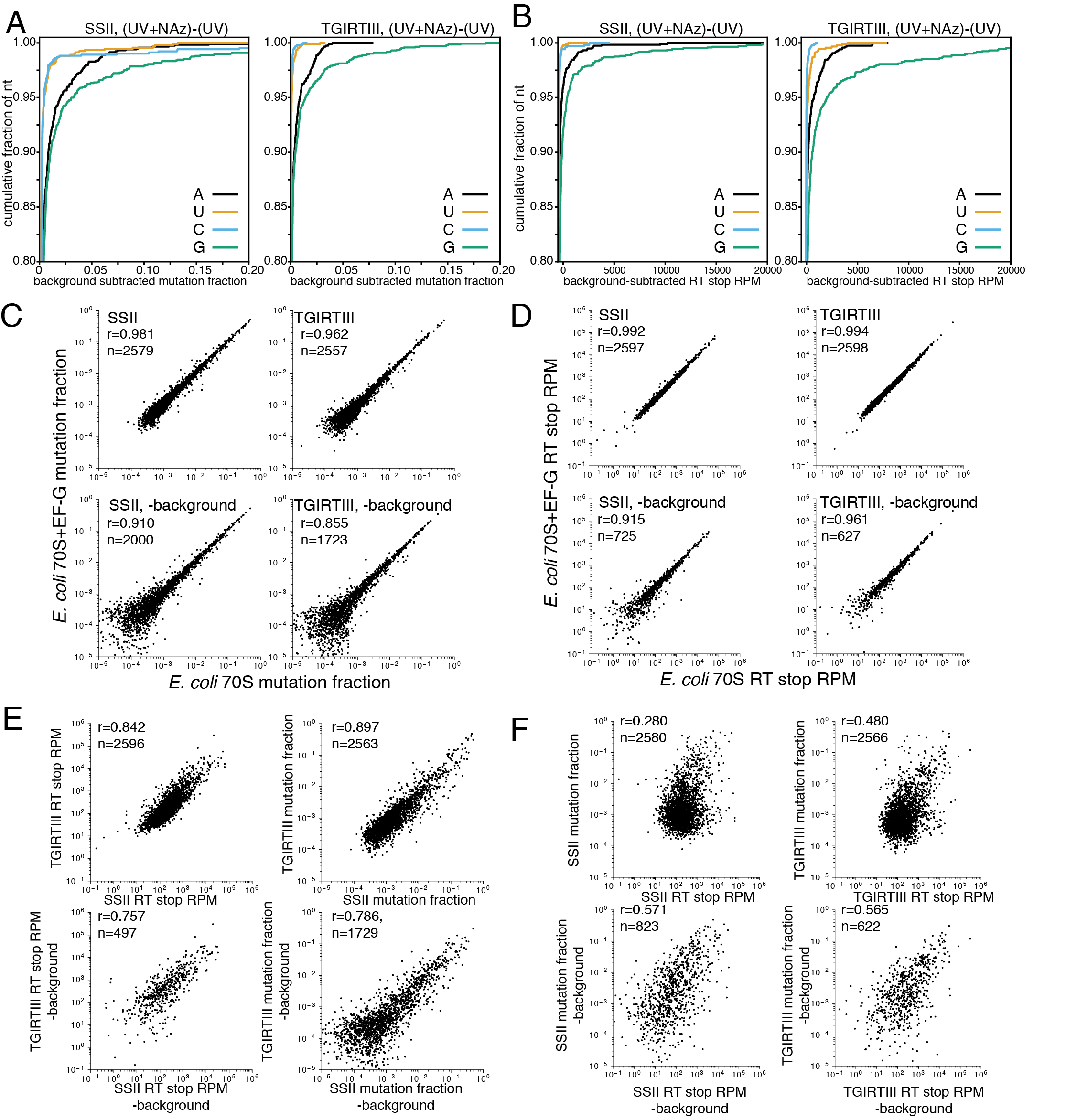


**Figure S3: Reproducibility of LASER-seq and effects of background subtraction.**

**A)** CDF of mutation fractions for each nucleotide, with UV-only control subtracted. **B)** CDF of background-subtracted RT stop RPMs for each nucleotide. **C)** Correlation of raw (top) or background-subtracted (bottom) mutation fractions for A and G nucleotides between libraries prepared from *E. coli* ribosomes with the same RT enzyme. These reactions are not exact replicates as half of them include preincubation with EF-G. The outlier nucleotides in these plots are explored in Figure 4. Spearman ρ, Pearson r and number of points are indicated on all correlation plots. **D)** Correlation of raw (top) or background-subtracted (bottom) RT stop RPMs for A and G nucleotides between libraries prepared from *E. coli* ribosomes with the same RT enzyme. **E)** Correlations between two different RTs for raw (top) or background subtracted (bottom) RT stop RPMs and mutation fractions. **F)** Correlation between raw (top) or background subtracted (bottom) RT stop RPMs and mutation rates for the same RT.


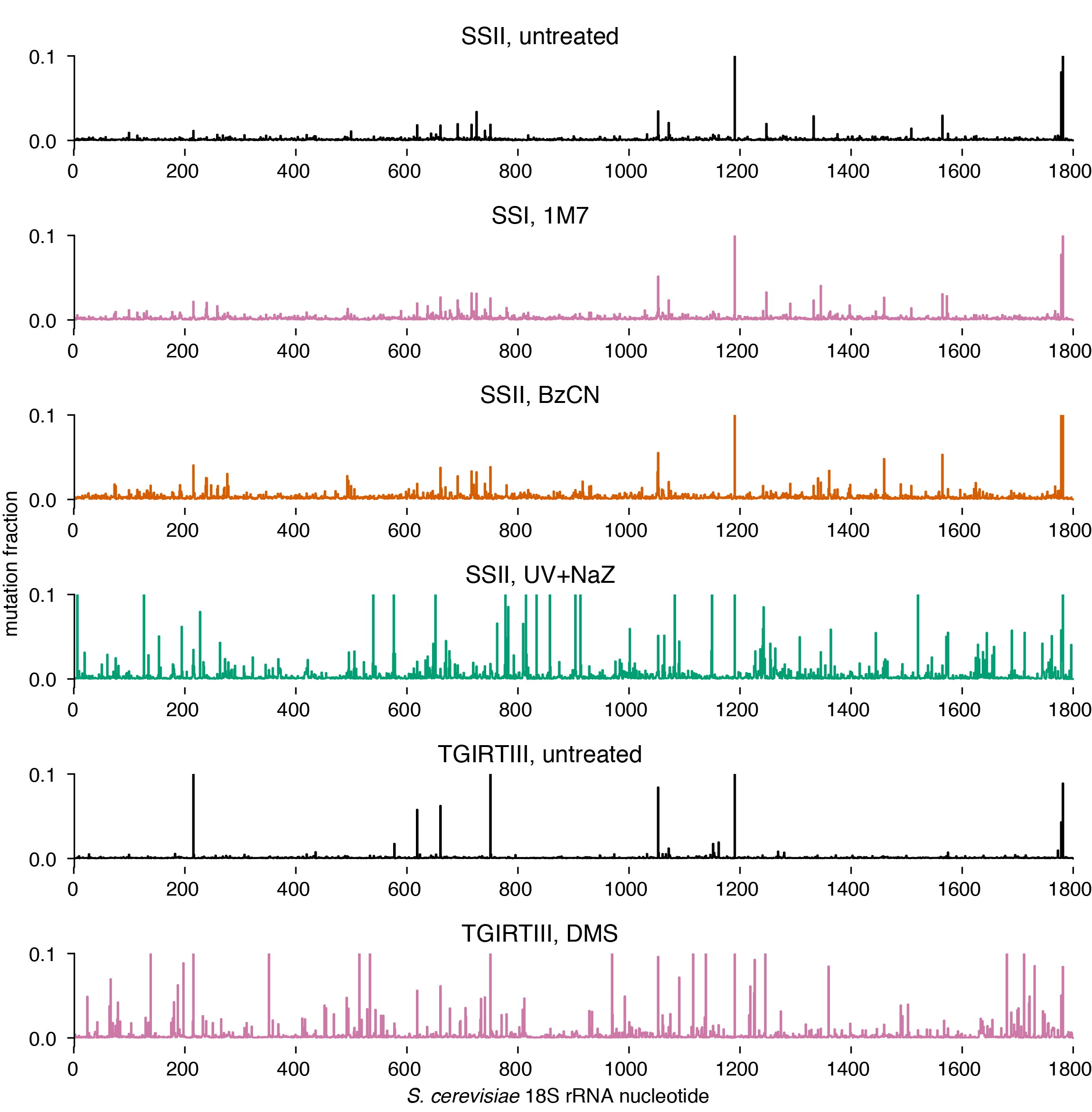


**Figure S4:**

Distribution of mutation rates across the *S. cerevisiae* 18S rRNA for MaP libraries probed with various reagents and reverse transcribed with SSII (1M7, BzCN, NAz) or TGIRTIII (DMS). DMS data from (McClary et al., 2017).


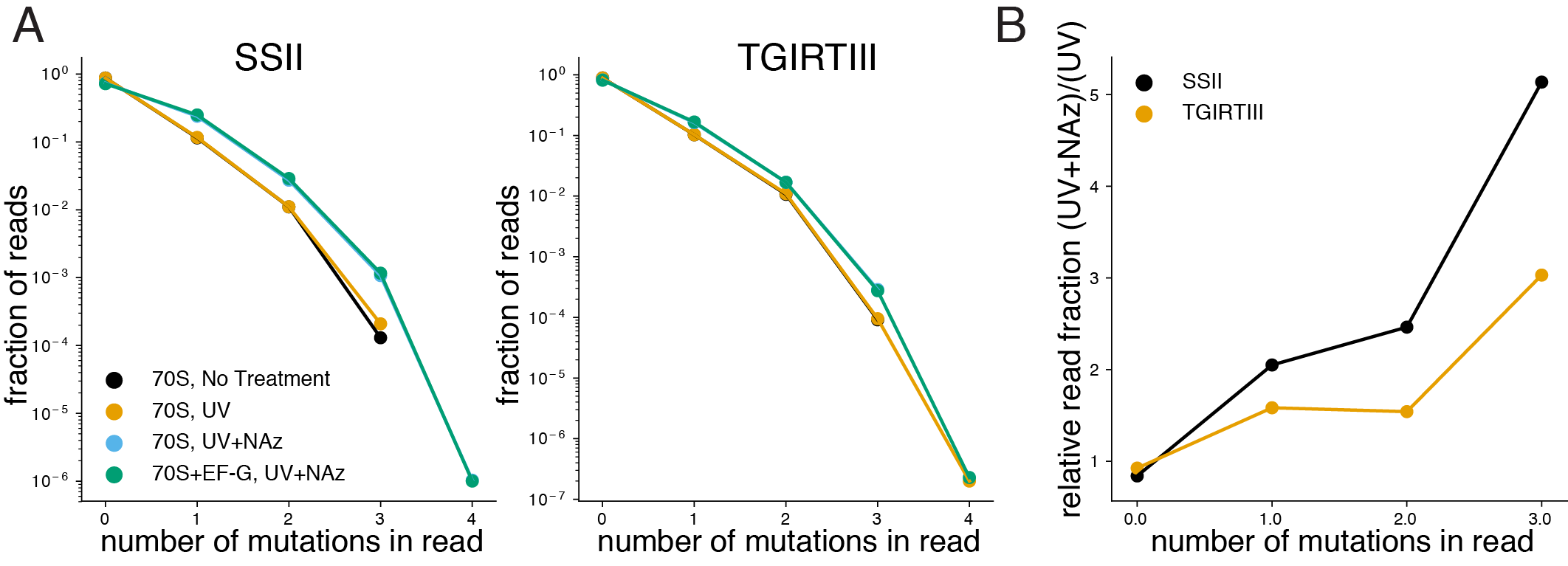


**Figure S5: Per-read mutation counts**

**A)** Fractions of reads containing indicated number of mutations for NAz-treated libraries or UV-only controls. **B)** NAz+UV / UV only ratio of read fractions containing each number of reads.


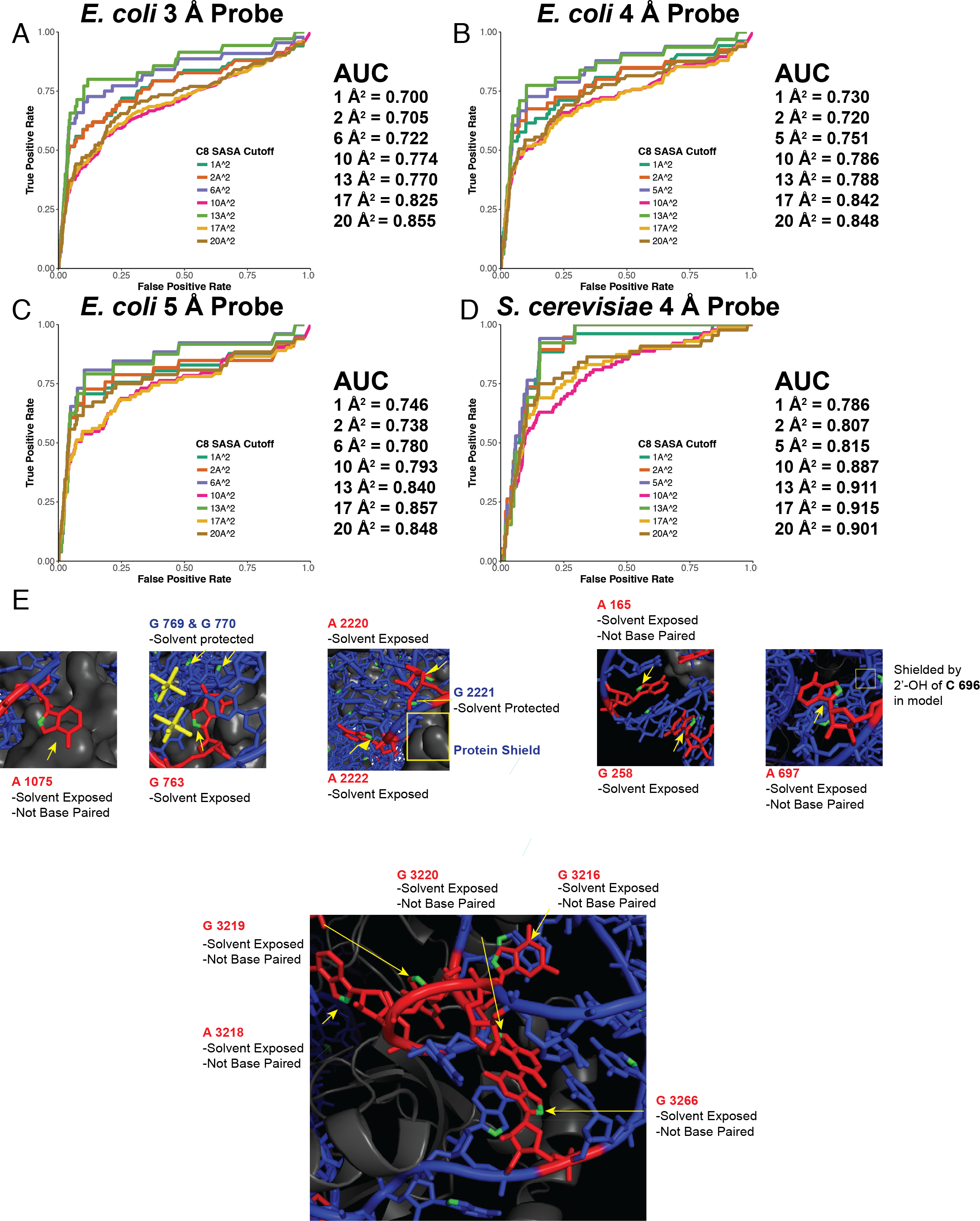


**Figure S6:**

ROC analysis for prediction of solvent-accessible nucleotides on *E. coli* using **A)** 3 Å probe radius, **B)** 4Å probe radius, **C)** 5Å probe radius, and **D)** *S. cerevisiae* 4Å probe radius. ROC curves were then calculated by setting different solvent accessibility limits for C8 of guanosine and adenosine residues. Slight increases in sensitivities were observed as the SASA cutoff for true positives was increased. **E)** Inspection of residues with high LASER-MaP reactivity, but low calculated SASA values in the yeast crystal structure (PDB ID 4V88). Highly reactive purines (labeled in red) were defined as those with mutation rate greater than 1% after background subtraction. C8 atoms are highlighted in green. Osmium (III) hexamine is labeled in yellow.


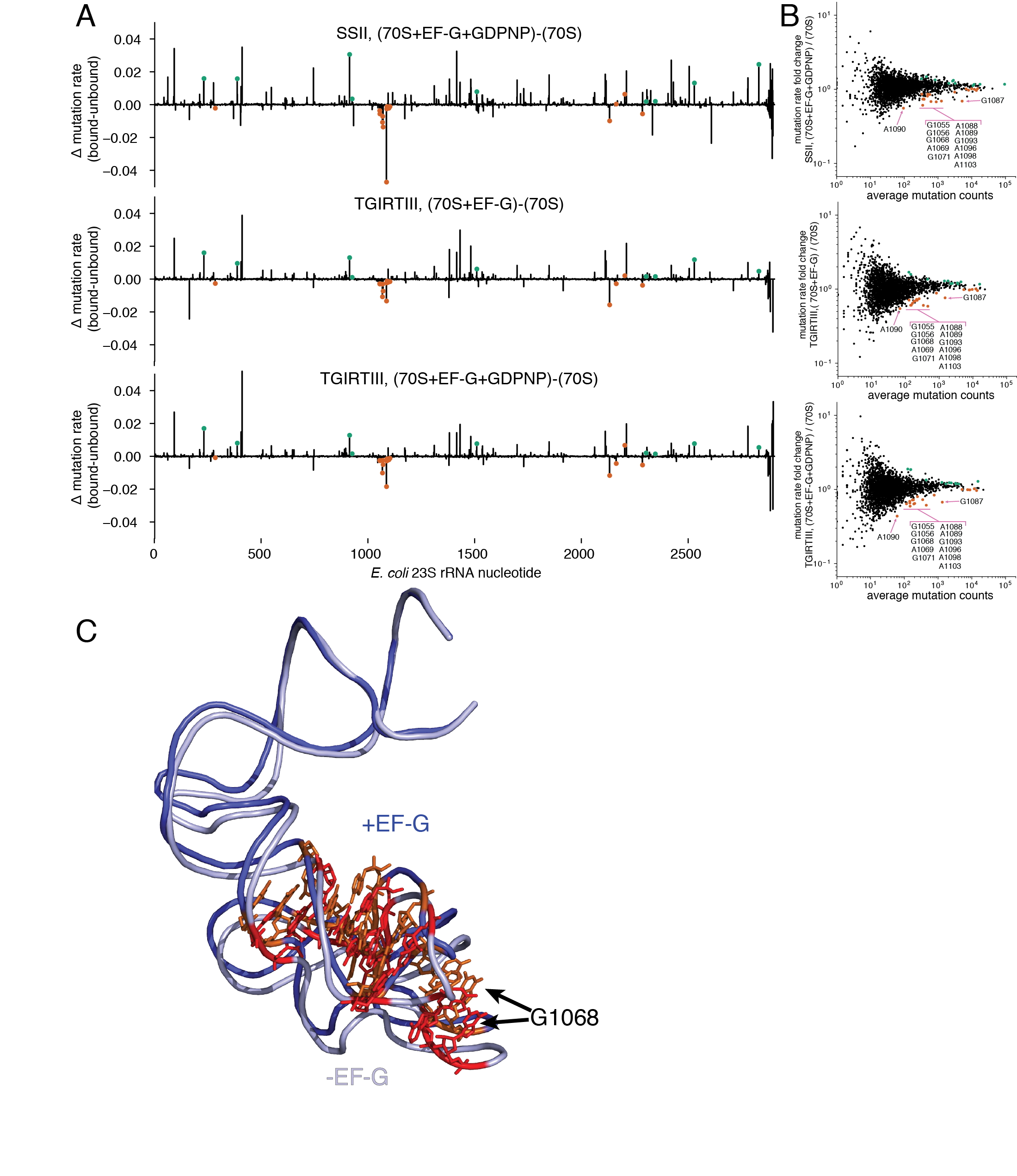


**Figure S7:**

**A)** Difference in LASER-MaP mutation fraction across the entire *E. coli* 23S rRNA, for EF-G bound ribosomes compared to unbound ribosomes. Nucleotides in the *E. coli* 23S rRNA with a statistically significant increase or decrease in all EF-G-treated samples are highlighted in green and orange, respectively**. B)** MA (log average vs log ratio) plot comparing the average number of mutations at rRNA nucleotides to their fold change in mutation fraction upon EF-G binding. **C)** The EF-G-bound GTPase-activation center (GAC, dark blue) of the *E. coli* ribosome viewed from the A site (PDB ID 3J9Z), with the unbound structure superimposed (PDB ID 4YBB) in light blue. The two structures were aligned in PyMol 2.0 (Schrödinger, LLC), based on the entirety of the 23S rRNA. Nucleotides protected by EF-G binding are highlighted on the bound and unbound structures in orange and red, respectively.

**
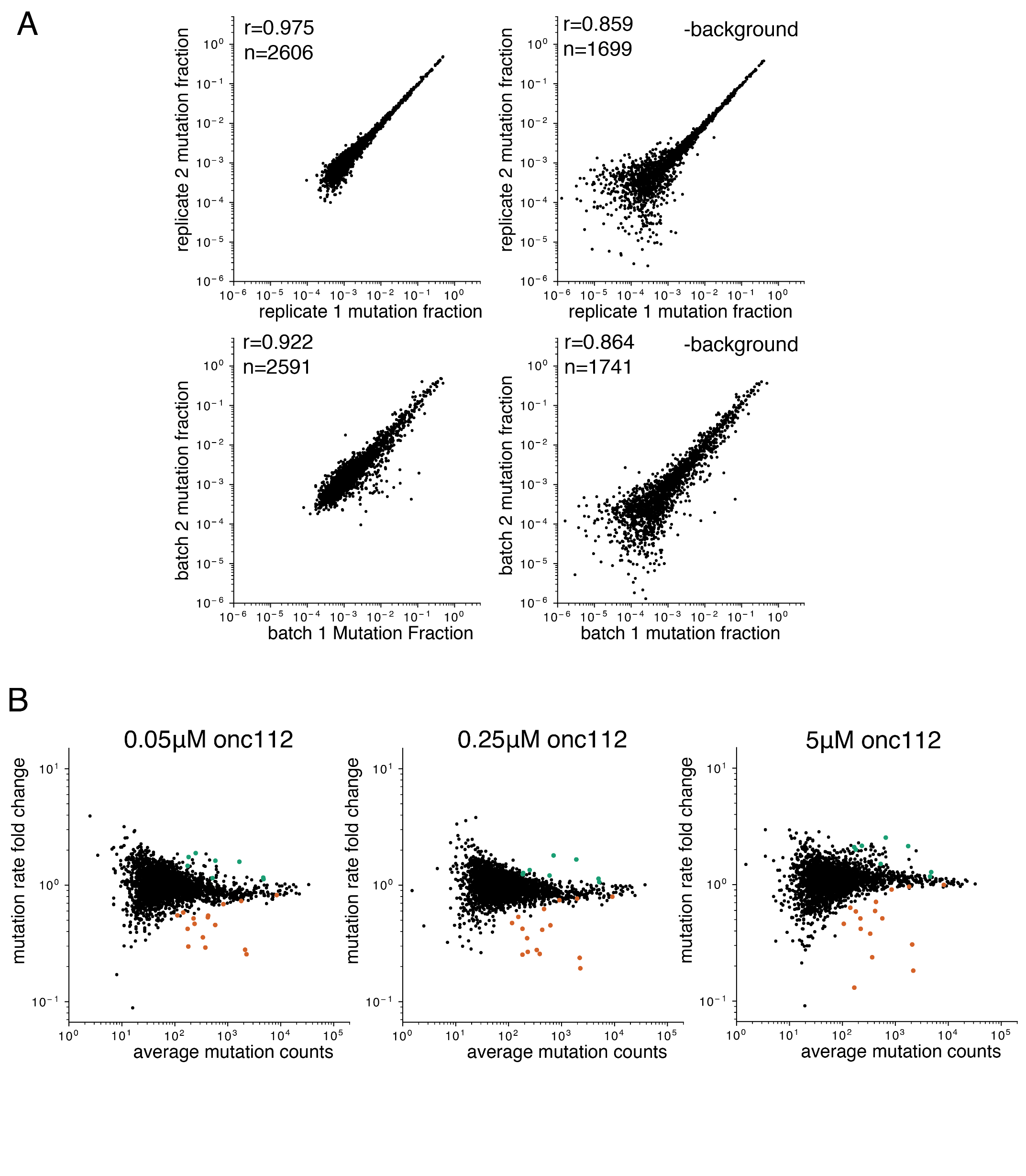
**

**Figure S8: Replicate reproducibility, and onc112**

**A)** Scatter plots of LASER-MaP mutation fractions for replicates from the same (top) or different (bottom) batches of reactions. **B)** MA (log average vs log ratio) plots comparing the average number of mutations at rRNA nucleotides to their fold change in mutation fraction upon onc112 binding.
